# Efficacy of multipoint versus conventional biventricular pacing in CRT: systematic review and meta-analysis of randomized trials

**DOI:** 10.1186/s43044-026-00761-4

**Published:** 2026-07-09

**Authors:** Tasneem Zanati Saeed, Ahmed Elkhouly, Waseem Emara, Nada Gamil, Imad Samman Tahhan, Bashar Abu-Mallouh, Rawan Fawzy, Basel Mohamed, Ahmed Adel Mohamed, Ahmed M. Gazer

**Affiliations:** 1 Faculty of Medicine, Assiut University, Assiut, Egypt; 2 Faculty of Medicine, Alexandria Univeristy, Alexandria, Egypt; 3 Faculty of medicine, Ain Shams University, Cairo, Egypt; 4 College of Medicine, Kafr Elsheikh University, Kafr Elsheikh, Egypt; 5 Department of Medicine, Wellington Regional Medical Center, Wellington, FL USA; 6 College of Medicine, Jordan University of Science and Technology, Irbid, Jordan; 7 Department of Public Health, Faculty of Medicine, Benha University, Benha, Egypt; 8 Faculty of Medicine, Zagazig University, Zagazig, Egypt

**Keywords:** Multipoint pacing, Heart failure, Heart failure with reduced ejection fraction, Cardiac resynchronization therapy, Echocardiographic response, Response rate

## Abstract

**Background:**

MultiPoint Pacing (MPP) may enhance the clinical and echocardiographic response to Cardiac Resynchronization Therapy (CRT), but randomized evidence remains inconsistent. The objective of this study is to compare the efficacy and safety of MPP versus conventional biventricular (BiV) pacing in patients with heart failure and reduced ejection fraction.

**Methods:**

We conducted a PROSPERO-registered (CRD420251033856) systematic review and meta-analysis of randomized controlled trials evaluating MPP versus BiV pacing. Outcomes included LVESV reduction, LVEF improvement, clinical response rate, super-response rate, and conversion to non-responder. Data were synthesized using random-effects models, and certainty was assessed with GRADE.

**Results:**

Six RCTs (n = 1,766) were included. MPP significantly improved LVEF (MD 5.16%, 95% CI 3.17–7.15; *P* < 0.0001), clinical response rate (RR 1.35, 95% CI 1.18–1.55; *P* < 0.0001), and super-response rate (RR 1.54, 95% CI 1.15–2.06; *P* = 0.004). The pooled LVESV analysis showed a nominally significant benefit with MPP, but this was accompanied by substantial heterogeneity and a prediction interval crossing zero, limiting confidence in a universal effect; the benefit appeared more consistent in CRT-naïve patients. No significant improvement was observed in non-responder conversion (RR 0.99). Overall certainty ranged from very low to moderate.

**Conclusion:**

Multipoint pacing is associated with signals of improved left ventricular reverse remodeling and higher clinical response rates compared with conventional biventricular pacing. However, the high heterogeneity and a 95% prediction interval crossing zero for LVESV reduction indicate that this effect is not consistent across all populations. MPP provides clinically meaningful benefits primarily in selected patient subgroups—particularly in CRT-naïve and responder populations—rather than serving as a universal rescue strategy for established non-responders.

**Supplementary Information:**

The online version contains supplementary material available at 10.1186/s43044-026-00761-4.

## Introduction

Cardiac resynchronization therapy (CRT) improves clinical outcomes in patients with moderate-to-severe heart failure with reduced ejection fraction (HFrEF) [1]. However, up to 40% of patients do not experience clinical improvement following conventional CRT. Prior studies indicated that patient-specific optimization of the LV lead position critically impacts CRT outcomes [2–4]. MultiPoint pacing (MPP)—via a quadripolar left ventricular lead- has emerged as a potential optimization strategy. MPP has been associated with improved hemodynamics, greater contractile synchrony, fewer reinterventions for lead dislodgement or phrenic nerve stimulation, and higher implantation success rates [5–7]. Experimental and early clinical studies suggest that MPP may outperform standard biventricular (BiV) pacing by recruiting a broader myocardial area, enhancing ventricular activation, and promoting more robust reverse remodeling [7]. In patients with significant distal conduction and contractility abnormalities, the MPP may improve clinical response and ventricular synchrony. Especially in cases of considerable electrical (> 30 ms) or mechanical dispersion (> 120 ms). In this difficult patient subgroup, it may be possible to more effectively overcome conduction delays by delivering stimulation from multiple LV sites [8].

Despite these promising mechanisms, the clinical evidence remains conflicting. While some studies reported improved remodeling and clinical response with MPP, larger randomized trials such as MORE-CRT failed to show incremental benefit over conventional BiV pacing. Emerging subgroup data also suggest that MPP may provide meaningful improvement only among carefully selected non-responders, highlighting the importance of patient selection [9, 10].

Therefore, the objective of this systematic review and meta-analysis was to synthesize evidence exclusively from randomized controlled trials (RCTs) to determine whether MPP is associated with improvement in echocardiographic and clinical outcomes compared with conventional BiV pacing.

## Methods

### General statement

This systematic review was conducted in accordance with the Cochrane Handbook for Systematic Reviews of Interventions [11] and reported in accordance with the Preferred Reporting Items for Systematic Reviews and Meta-Analyses (PRISMA) guidelines [12]. The protocol was registered in PROSPERO (CRD420251033856).

### Eligibility criteria

We included only RCTs that enrolled patients aged 18 years or older with heart failure with HFrEF defined as a left ventricular ejection fraction (LVEF) of 35% or less and a clinical indication for CRT, including both CRT-naïve patients and those who were non-responders to BiV pacing, in which the intervention was MPP with any programming strategy (a proprietary pacing mode where two LV electrodes fire simultaneously or with a programmable delay via a quadripolar lead) and the comparator was biventricular pacing (using a bipolar lead or LV quadripolar lead programmed for single-site pacing). We excluded case series, case reports, observational studies, editorials, comments, letters, reviews, book chapters, and non-English language studies.

### Search strategy

We systematically searched PubMed, Scopus, Cochrane Library, and Web of Science from inception to May 2025. The complete search strings are provided in Supplementary Table S1.

### Study selection and data extraction

Five authors independently screened the studies for possible inclusion using Rayyan and Google Sheets [13]. Five authors extracted the summary of included studies, detailing study ID, study arms, country, study design, included population, number of participants in each arm, implanted lead location, pacing mechanism, MPP subgroups, study duration, follow-up duration, primary outcomes for each study, key findings, and subgroup key findings. Also, we extracted population characteristics, including age, male prevalence, ischemic heart disease, hypertension, diabetes, body mass index (BMI), QRS width, LVEF, left ventricular end-diastolic volume (LVEDV), left ventricular end-systolic volume (LVESV), and NYHA class. (W.E) reviewed all extracted data. Any conflicts were solved by (T.Z., A.G.).

### Risk of bias assessment

The risk of bias for the included randomized clinical trials was independently assessed by two reviewers using the Cochrane Risk of Bias tool for randomized trials (ROB 2) [14] who evaluated each study across the following five domains: 1) Bias arising from the randomization process, 2) Bias arising from the intended interventions, 3) Bias due to missing outcome data, 4) Bias in the measurement of the outcome, and 5) Bias in the selection of the reported results. They assessed it following the algorithms and signalling questions described in the official ROB-2 manual. The conflict was solved through discussion. The overall risk of bias for each study was determined based on domain-level rating as “Low risk,” “Some concerns,” or “High risk.” We generated a traffic light plot to summarize domain-level and overall judgments across all studies.

### Outcome definitions/measures

For dichotomous outcomes (response and super-response), we accepted study-specific definitions but ensured conceptual consistency across trials, as most studies defined response as ≥ 15% reduction in LVESV and super-response as ≥ 30% reduction in LVESV. When composite definitions included additional clinical parameters (e.g., NYHA class), these were included as reported to preserve trial-level validity. Because response definitions varied across trials—some strictly echocardiographic and others incorporating clinical endpoints—this variability was considered a potential source of indirectness and was accounted for in the GRADE assessment. Negative values for LVESV change represent greater reduction favoring MPP, whereas positive values for LVEF change represent greater improvement favoring MPP.

### Data synthesis and statistical analysis

Meta-analyses were performed using the RevMan software web [15], applying a random-effects model. Continuous outcomes (LVESV%, LVEF absolute points) were synthesized using Mean Difference (MD) with 95% Confidence Intervals (CI). Dichotomous outcomes (response rate, super-response rate, and conversion rate) were synthesized using risk ratios (RR) with 95% CIs. Statistical heterogeneity was assessed using the I^2^ statistic.

To provide a more robust interpretation of the treatment effect in the presence of heterogeneity, 95% prediction intervals (PI) were calculated for the primary outcome to estimate the potential range of true effects in future clinical settings. Sources of heterogeneity were further explored through subgroup analyses stratified by baseline patient population (primarily CRT-naïve vs. prior non-responders/mixed) and sensitivity analyses to assess the robustness of the findings.

Additional outcomes were assessed qualitatively through narrative review due to inconsistent reporting formats, insufficient data across studies for pooling, or significant differences in study populations and measurement timeframes. These included proportion of patients improving by ≥ 1 NYHA functional class, all-cause or cardiac mortality, heart failure hospitalizations (HFH), mean change in Left Ventricular End-Diastolic Volume (LVEDV), mean change in QRS duration (QRSd), mean change in Quality of Life scores (e.g., Minnesota Living with Heart Failure Questionnaire [MLHFQ], EQ-5D), mean change in 6-Minute Walk Test (6MWT) distance, and Clinical Composite Score (CCS) as reported in individual trials. Publication bias was evaluated in the GRADE assessment [16].

## Results

### Study selection

**Fig. 1:**
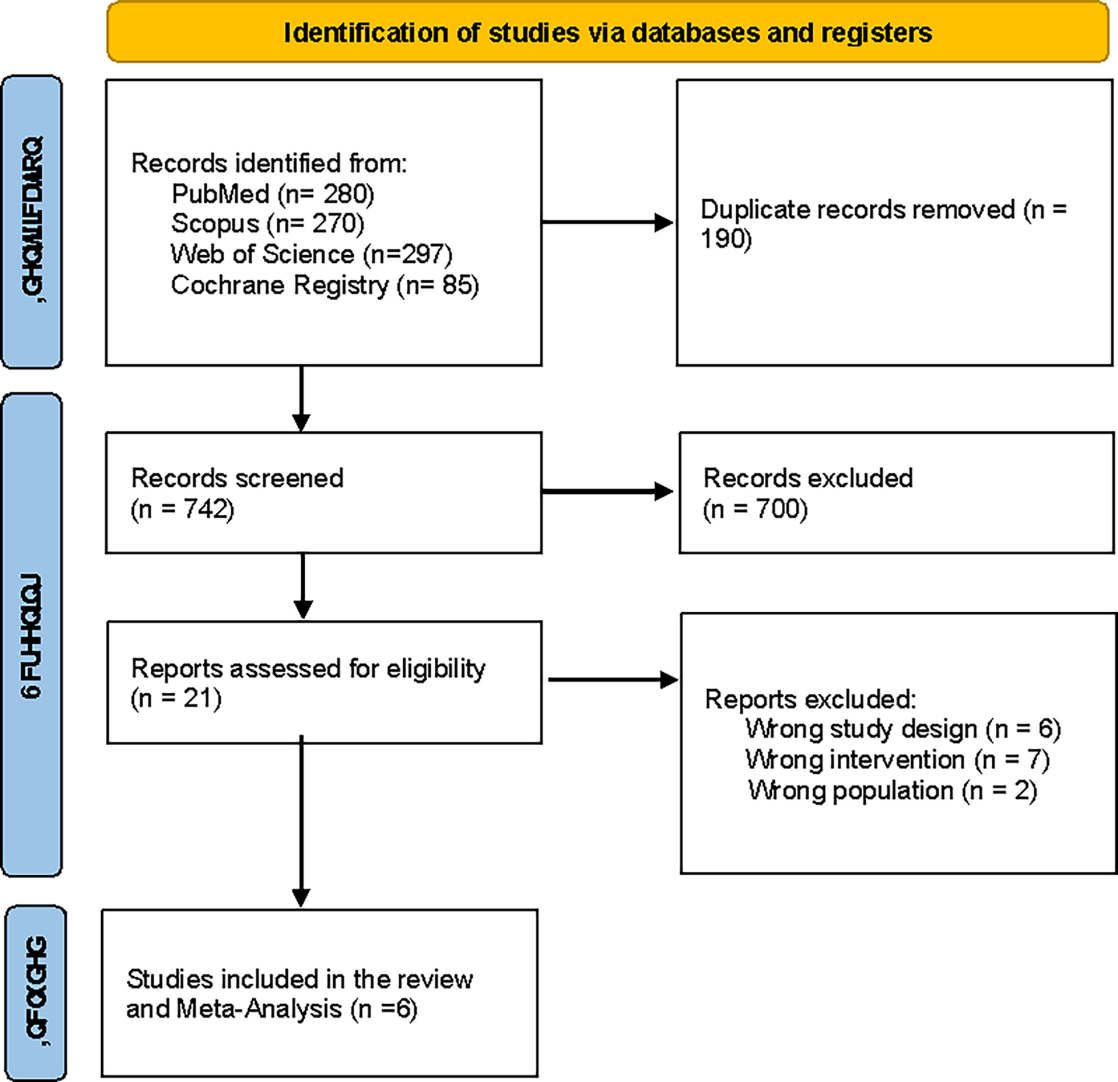
PRISMA flow diagram of study selection process. The flow diagram outlines the methodical search and selection procedure for randomized controlled trials that compare biventricular (BiV) and multipoint pacing (MPP) in patients with heart failure with reduced ejection fraction (HFrEF)

A total of 932 records were identified through database searching. After removing duplicates and screening full text for eligibility, six studies were included in the final analysis Fig. 1.

### Characteristics of included studies

The included studies were published between 2015 and 2023 and enrolled 1,766 participants (852 MPP; 914 BiV). Detailed characteristics of each study are provided in Tables 1 and 2, and the outcomes summary is presented in Table 3.

### Overall bias assessment

Risk of bias was evaluated for all six included randomized trials using the Cochrane ROB-2 tool. Overall, three studies—Gu et al. [5], Marques et al. [3], and Pappone et al. [1]—were judged as having a *Low risk of bias,* with adequate randomization, minimal attrition, blinded outcome assessment, and complete reporting. Two studies—Almusaad et al. [9] and Leclercq et al. [7]—raised concerns about the overall risk of bias, primarily due to limited reporting of randomization procedures, the absence of protocol registration, or incomplete echocardiographic follow-up. One study—Varma et al. [2]—which was a secondary analysis of a parent RCT, was rated as *high risk of bias*, mainly due to substantial missing outcome data and selective reporting in post hoc subgroup analyses.

**Fig. 2:**
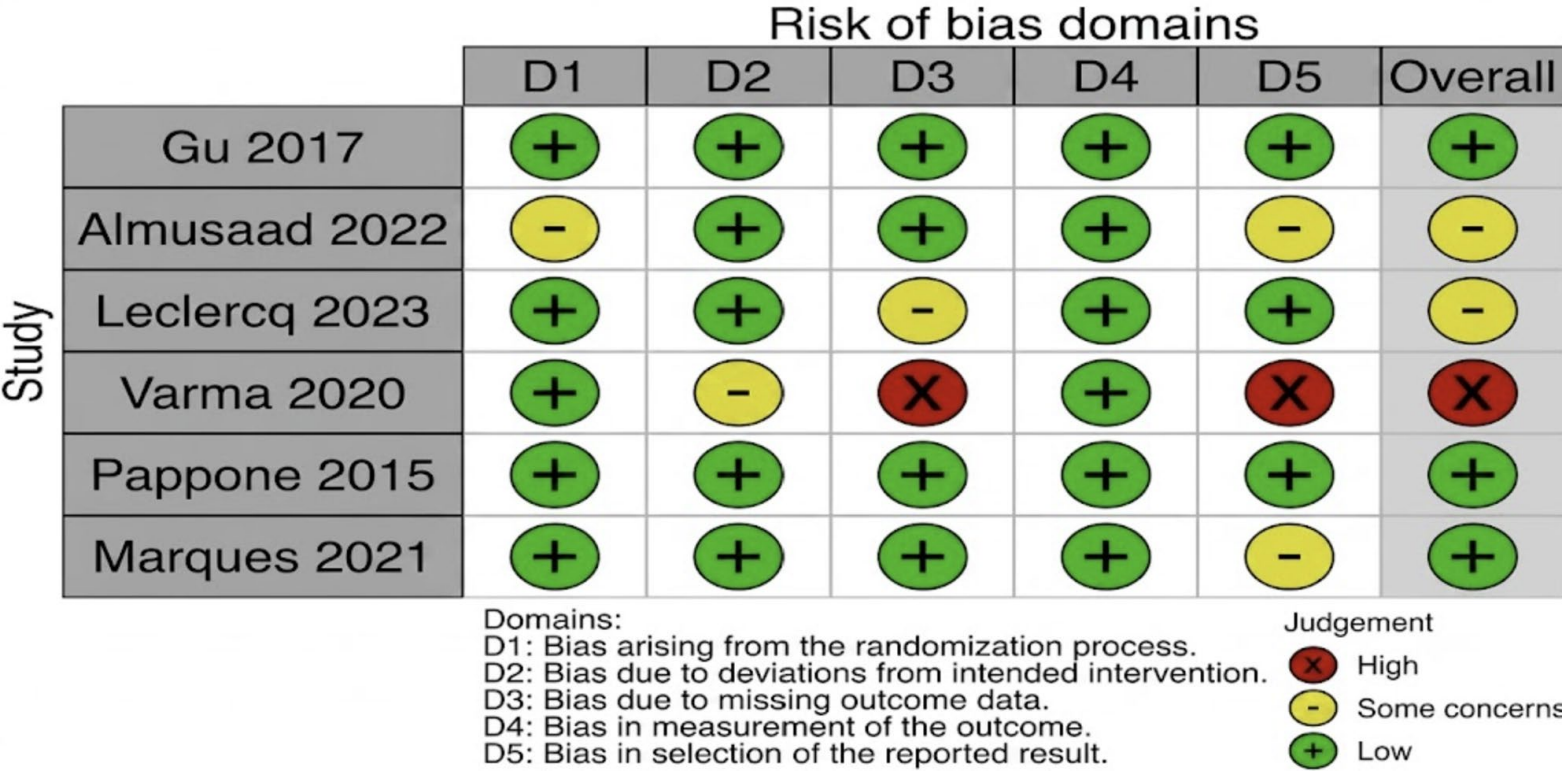
Risk of Bias Summary (Cochrane RoB-2). Summary plot illustrating the domain-level and overall risk of bias judgments for the six included randomized controlled trials. Assessments were conducted using the Cochrane Risk of Bias 2.0 tool. The overall risk rating reflects the highest risk level identified across the five domains for each study

Across domains, most trials showed a low risk of deviations from intended interventions and outcome measurement, reflecting the device-driven nature of CRT programming and the frequent use of blinded echocardiographic core laboratories. Missing outcome data were low, except in studies with extended imaging follow-up. Concerns related to selective reporting were limited to studies without prospective registration. These assessments indicate that, while methodological limitations exist, the overall body of evidence is of acceptable quality for evaluating the comparative effects of the included pacing strategies. Nevertheless, several trials were small, single-center, and unblinded with respect to device programming; therefore, despite the use of echocardiographic core laboratories, some residual performance and detection bias cannot be entirely excluded. Figure 2 presents the summary plot for ROB-2, and Table S2 supports the risk-of-bias assessment judgment.

**Table 1:** Characteristics of studies included in the analysis

Study ID	Arms	Design and Country	Population	Lead’s location	Pacing mechanism	Duration of the study	Follow-up	Primary outcome	Key findings	Key subgroup findings
Almusaad et al. [9]	MPP (73)	RCT; Multinational (KSA, Oman, Iraq, Bahrain, Egypt, UAE, Kuwait)	De-novo CRT-D; ≥ 18y	RA: Appendage. RV: Apex LV: Medial (76%), Postero-lateral (59%), or Lateral (39%)	Vectors: Earliest (LV1) & Latest (LV2) RV-LV conduction Delays: LV1➔LV2➔RV (5ms)	2019–2020	6 mon	Response rate to CRT at 6 months (by changes in LV end-systolic volume (ΔESV) and LV ejection fraction (ΔEF)	Responders' rate in MPP (68.5%) vs in BiV (50.7%). *P* = 0.04	NA
	BiV (69)				Vector: Single LV Delay: LV➔RV, 10 ms)					
Marques et al. [3]	MPP (24)	RCT [single-blind study]; Portugal	≥ 18y with HF & ESC CRT indication; Sinus rhythm (No AF ≤ 30 days)^3^	RA: Appendage. RV: Apex/Septum. LV: Coronary Sinus	Vectors: 2 LV cathodes. Delays: LV1➔ LV2 (5 ms) ➔ RV (5 ms) Criteria: Threshold ≤ 4.5 V & No PNS at 1.0 V margin	Feb. 2017–Feb. 2019	6 and 12 mon	Efficacy (determined by 6–12-month changes in LVESV and LV ejection fraction (LVEF))	1- LVESV change: -8.3% MPP vs -10.3% BiV *p* = 0.047. 2- LVEF change: 7.7% MPP vs 1.8 BiV p 0.008	NA
	BiV (25)				Vector: Single LV cathode (Latest RV-LV activation) Safety: Threshold ≤ 4.5 V & No PNS at 1.0 V margin					
Pappone et al. [1]	MPP (19)	RCT; Italy	≥ 18y with ESC/EHRA CRT indication; able to consent	RA: Appendage. RV: Apex/Septum. LV: Coronary Sinus	Vectors: Dual LV (LV1 & LV2) Delays: Programmable LV1➔LV2 and LV2➔ RV intervals	April to November 2012	12 mon	CRT response (defined by being alive status and ESV decrease > = 15% relative to baseline)	1- LVESV change: -25% MPP, -17% BiV, *p* = 0.03 2- LVEF change: 15% MPP, 5% BiV, *p* < 0.01	NA
	BiV (21)				Conventional BiV (Single LV vector)					
Gu et al. [5]	MPP (26)	RCT; China	≥ 18y; NYHA II–IV; LVEF ≤ 35%; QRS ≥ 120 ms	RA: Appendage. RV: Apex/Septum. LV: Quadripolar	MPP (10 LVPC options)	6 months	3 and 6 mon	CRT response (NYHA class improvement > = LVESV reduction ≥ 15%)	1-MPP 80.8%, BiV 68% responders at 6 months 3-Greater LVEF increase (+ 12.7% MPP VS + 7.8% BiV) 3-LVESV reduction (− 26.9% MPP VS − 17.2% BIV)	NA
	BiV (25)			RA/RV: Standard. LV: Conventional bipolar lead	Conventional BiV pacing (tip-ring)					
Varma et al. [2]	MPP (43)	RCT; USA	≥ 18y receiving CRT; LV enlargement (LVEDVI > 1.1 mL/cm)	The LV lead was placed on the coronary sinus	MPP-AS: ≥ 30 mm separation; 5 ms delay	9 Months	3 and 9 mon	CCS responder rate (NYHA class, HF events, mortality)	92% MPP-AS responders vs 65% BiV, 0% HF events post-randomization	MPP-AS is effective only in patients with LV enlargement (LVEDVI > Median)
	BiV (188)				Single-site LV stimulation via quadripolar lead					
Leclercq et al. [7]	MPP (541)	RCT; Multinational (France, Switzerland, Netherlands, UK, Germany, Italy, Spain, KSA, Canada, USA)	≥ 18y; LVEF ≤ 35%; 6-mo BiV Non-responders (ΔLVESV < 15%)	RA: Appendage. RV: Apex/Septum. LV: Coronary Sinus	Simultaneous dual-site LV; spacing ≥ 30 mm (MPP-AS)	12 months	6 mon	Non-responding to responder conversion (> = 15% reduction in LVESV) from 6 to 12 months)	No significant difference in primary endpoint: MPP arm: 29.4% became responders, BiV arm: 30.4% became responders. No significant difference in mean % LVESV reduction or NYHA improvement	Overall Conversion: 29.4% (MPP) vs 30.4% (BiV) (*P* = 0.92) > 98% Pacing Subgroup: MPP superior in ITT (43% vs 32%, *P* = 0.043) and As-treated analysis (44% vs 31%, *P* = 0.036)
	BiV (570)				Single LV cathodal pacing					

BiV, Biventricular pacing; CRT-D, Cardiac resynchronization therapy defibrillator; HF, Heart failure; ITT, Intention-to-treat; LVEDVI, Left ventricular end-diastolic volume index; LVESV, Left ventricular end-systolic volume; MPP, MultiPoint pacing; MPP-AS, MultiPoint pacing with anatomical separation; PNS, Phrenic nerve stimulation; RA/RV, Right atrial/right ventricular; RCT, Randomized controlled trial

**Table 2:** Baseline characteristics of participants included in the study

Study ID	Arms	Age, year	Male, n	Ischemic heart disease	Hypertension	Diabetes	BMI, kg/m^2^	QRS width (ms)	EF, %	LVEDV (ml)	LVESV (ml)	NYHA class, n (%)		
												II	III	IV
Almusaad et al. [9]	MPP	61.27 ± 12.93	48	26 (35.6%)	47 (64.4%)	35 (47.9%)	NA	160.77 ± 16.87	26.9 ± 9.45	202.33 ± 97.56	146.33 ± 77.9	11 (15.1%)	57 (78.1%)	5 (6.8%)
	BiV	59.57 ± 11.29	48	21 (30.4%)	34 (49.3%)	39 (56.5%)		159.33 ± 15.14	26.63 ± 8.18	199.6 ± 65.27	144.3 ± 67.39	16 (23.2%)	50 (72.5%)	3 (4.3%)
Marques et al. [3]	MPP	64.5 ± 10.2	13	5 (20.8%)	20 (83.3%)	12 (50.0%)	NA	170 ± 20.49	28.7 ± 6.9	210.5 ± 81.9	153.9 ± 73.2	17 (70.8%)	7 (29.2%)	NA
	BiV	66.5 ± 11.6	15	4 (16%)	20 (80.0%)	10 (40.0%)		162.67 ± 18.87	29.6 ± 6.9	189.4 ± 53.9	134.7 ± 47.1	17 (68.0%)	8 (32.0%)	
Pappone et al. [1]	MPP	65 ± 9	16	9 (47%)	NA	NA	NA	154 ± 17	27 ± 7	248 ± 62	182 ± 56	NA	19 (100%)	NA
	BiV	67 ± 8	15	8 (38%)				152 ± 18	30 ± 6	237 ± 124	169 ± 107		21 (100%)	
Gu et al. [5]	MPP	55.8 ± 11.1	19	4 (15.4%)	9 (34.6%)	7 (26.9%)	NA	161.8 ± 16.3	28.2 ± 6.6	262.0 ± 81.1	173.3 ± 68.8	10 (38.5%)	15 (57.7%)	1 (3.8%)
	BiV	58.7 ± 9.3	17	4 (16%)	10 (40%)	5 (20%)		159.7 ± 13.0	28.7 ± 6.7	272.0 ± 82.3	185.6 ± 73.1	9 (36%)	16 (64%)	1 (4%)
Varma et al. [2]	MPP	LVEDVI > Median: 65 ± 11 years and LVEDVI < Median: 68 ± 10 years	LVEDVI > Median: 17/24(70.8%) and LVEDVI < Median: 11/19(57.9%)	LVEDVI > Median: 62.5 and LVEDVI < Median: 57.9	NA	NA	NA	LVEDVI > Median: 153 ± 22 and LVEDVI < Median: 158 ± 27	LVEDVI > Median: 25 ± 6 and LVEDVI < Median: 32 ± 7	LVEDVI > Median: 241 ± 51 and LVEDVI < Median: 152 ± 33	LVEDVI > Median: 181 ± 44 and LVEDVI < Median: 106 ± 28	LVEDVI > Median: 41.7 and LVEDVI < Median: 15.8	LVEDVI > Median: 37.5 and LVEDVI < Median: 52.6	LVEDVI > Median: 12.5 and LVEDVI < Median: 5.3
	BiV	LVEDVI > Median: 65 ± 11 and LVEDVI < Median: 70 ± 9	LVEDVI > Median: 78/94(83.0%) and LVEDVI < Median: 56/94(59.6%)	LVEDVI > Median: 51.1 and LVEDVI < Median: 50.0				LVEDVI > Median: 159 ± 22 and LVEDVI < Median: 150 ± 18	LVEDVI > Median: 26 ± 7 and LVEDVI < Median: 33 ± 8	LVEDVI > Median: 252 ± 58 and LVEDVI < Median: 146 ± 27	LVEDVI > Median: 187 ± 52 and LVEDVI > Median: 99 ± 23	LVEDVI > Median: 34.0 and LVEDVI < Median: 26.6	LVEDVI > Median: 53.2 and LVEDVI < Median: 61.7	LVEDVI > Median: 2.1 and LVEDVI < Median: 2.1
Leclercq et al. [7]	MPP	68 ± 10	438 (81%)	287 (53%)	NA	NA	NA	155 ± 26	26 ± 8%	NA	164 ± 68	346 (64%)	104 (19.2%)	NA
	BiV	68 ± 11	443 (77.7%)	291 (51.1%)				153 ± 25	26 ± 7%		163 ± 68	353 (61.9)	105 (18.4%)	

Include extracted population characteristics, including age, male prevalence, ischemic heart disease, hypertension, diabetes, body mass index (BMI), QRS width, LVEF, left ventricular end diastolic volume (LVEDV), left ventricular end systolic volume (LVESV), and NYHA class

**Table 3:** Analyzed outcomes and their definitions

Study ID	Outcome	MPP	BiV	Tool	Time of evaluation
Almusaad et al. [9]	LVESV reduction	−25.0% [−37.2%, −11.5%] (patients: 73)	−15.3% [−31.3%, −3.3%] (patients: 69)	Echo exam	6 months
	LVEF improvement	11.9% [5.5%, 19.7%] (patients: 73)	8.6% [3.7%, 16.9%] (patients: 69)	Echo exam	6 months
	Response Rate	68.5% (patients: 50/73)	50.7% (patients: 35/69)	ESV ≥ 15% reduction	6 months
	Super-response Rate	39.7% (patients: 29/73)	27.5% (patients: 19/69)	ESV reduction ≥ 30%	6 months
Marques et al. [3]	LVESV reduction	−8.3 ± 23% (patients:24)	−10.3 ± 35.8% (patients:25)	Echo exam	12 months
	LVEF improvement	7.7 ± 5.4% (patients: 24)	1.8 ± 8.3% (patients: 25)	Echo exam	12 months
	Super-response Rate	86.4% (patients: 19/22)	56% (patients:14/25)	ESV reduction ≥ 30%	12 months
Pappone et al. [1]	LVESV reduction	−25% [−39%, −20%] (patients:19)	−18% [−25%, 2%] (patients: 21)	Echo exam	12 months
	LVEF improvement	15% [8%, 20%] (patients:19)	5% [−1%, 8%] (patients: 21)	Echo exam	12 months
	Response Rate	76% (patients: 16/21)	57% (patients: 12/21)	ESV reduction ≥ 15%	12 months
	Super-response Rate	33% (patients: 7/21)	14% (patients: 3/21)	ESV reduction ≥ 30%	12 months
	Non-responders becoming responders	3 of 5 (60%)	5 of 11 (45%)	Responders’ classification based on Echocardiography (ESV reduction)	Between 3 and 12 months
Gu et al. [5]	LVESV reduction	−26.9 ± 13.8% (patients: 26)	−17.2 ± 13.3% (patients: 26)	Echo exam	3–6 months
	LVEF improvement	12.7 ± 8.0% (patients: 26)	7.8 ± 6.3% (patients: 26)	Echo exam	3–6 months
	Response Rate	80.8% (patients: 21/26)	68% (patients:17/25)	Alive, NYHA improv. ≥ 1, ESV red. ≥ 15%	3–6 months
	Non-responders becoming responders	50% (patients: 4/8)	22.2% (patients: 2/9)	Number from non-responders	6 months
Varma et al. [2]	Response Rate	63% [LVEDVI > Median] (patients: 22/24)	48% [LVEDVI > Median] (patients: 61/94)	↑LVEF ≥ 5% and ↓LVESV ≥ 10%	9 months
Leclercq et al. [7]	LVESV reduction	2 ± 26% (patients: 541)	4 ± 23% (patients: 570)	Echo exam	12 months
	Non-responders becoming responders	29.4% (patients: 159/541)	30.4% (patients: 173/570)	Echo exam	12 months

Definitions and reported outcomes across included studies

**Table 4:** Summary of Findings: MultiPoint Pacing (MPP) vs Biventricular Pacing (BiV) in CRT (GRADE Assessment)

Outcome	No. of RCTs (participants)	Effect (95% CI)	Certainty of evidence	Reasons for downgrading
LVESV Reduction (% change)	5 RCTs (n = 1,394)	MD 7.57% (0.76 to 14.39)	**Very low**	Serious RoB (some concerns), Serious heterogeneity (I^2^ = 77%), Serious imprecision (PI crosses 0)
LVEF Improvement (absolute % points)	4 RCTs (n = 283)	MD 5.16% (3.17 to 7.15)	**Moderate**	RoB (some concerns in 3/4 studies), no imprecision, moderate heterogeneity
Composite Clinical Response	4 RCTs (n = 353)	RR 1.35 (1.18–1.55)	**Moderate**	Downgraded for some concerns in RoB, consistent effect, no imprecision, no heterogeneity
Super-Response Rate	3 RCTs (n = 231)	RR 1.54 (1.15–2.06)	**Low**	Small sample, some concerns in RoB, borderline imprecision
Non-Responder to Responder Conversion	3 RCTs (n = 1,144)	RR 0.99 (0.83–1.18)	**Very low**	High heterogeneity across populations, serious imprecision (CI includes meaningful harm/benefit), RoB concerns

This table presents the GRADE assessment of the certainty of evidence for key echocardiographic and clinical outcomes comparing MultiPoint Pacing (MPP) with conventional biventricular (BiV) pacing. Certainty ratings were based on risk of bias, inconsistency, indirectness, imprecision, and publication bias according to the GRADE methodology. The pooled effect estimates were derived from randomized controlled trials only. The certainty of the evidence was downgraded when substantial methodological concerns, heterogeneity, or imprecise confidence intervals were present. Higher certainty indicates greater confidence that the estimated effect is close to the true effect

### Main outcomes

#### LVESV reduction

**Fig. 3:**
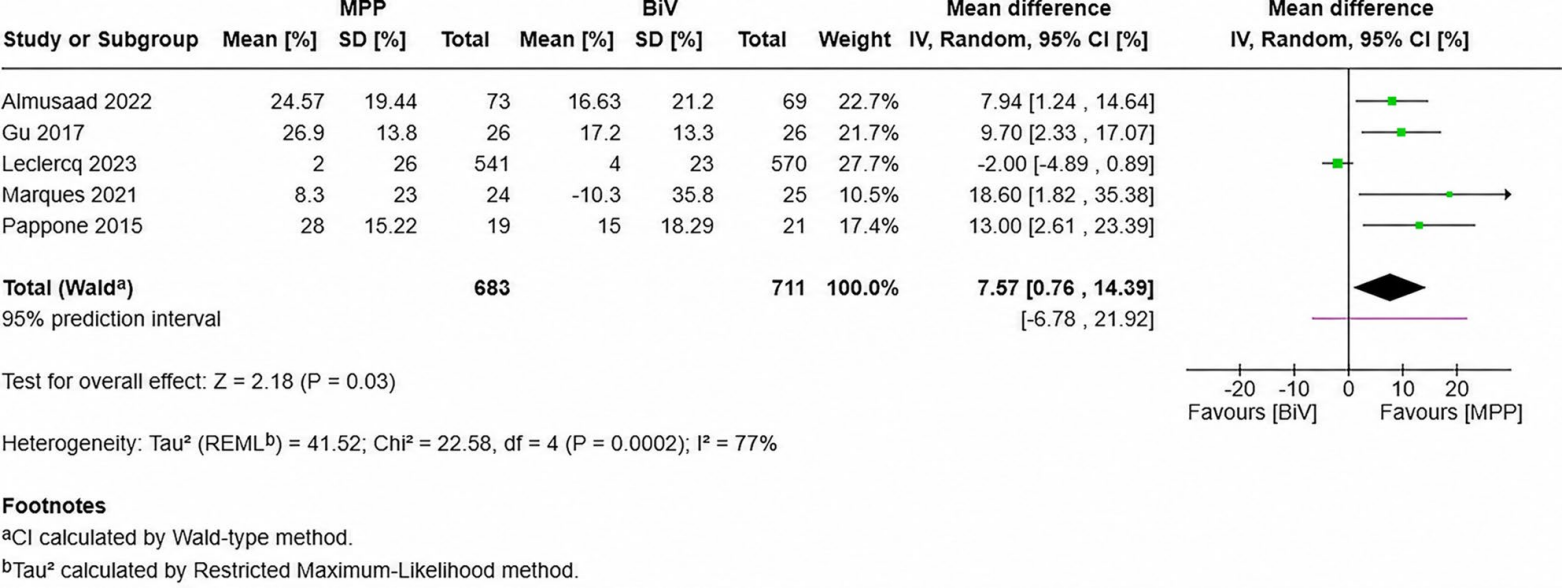
Forest plot of the primary meta-analysis comparing the percentage reduction in Left Ventricular End-Systolic Volume (LVESV) between MultiPoint Pacing (MPP) and Conventional Biventricular Pacing (BiV). The forest plot displays the mean difference (MD) in the percentage reduction of LVESV from baseline to follow-up. The purple horizontal line at the bottom represents the 95% prediction interval (−6.78 to 21.92), indicating the range in which the true effect of a future study is expected to fall

Five randomized controlled trials (n = 1,394) reported data on the percentage reduction in Left Ventricular end-Systolic Volume (LVESV) from baseline, comparing multipoint pacing (MPP) (n = 683) to conventional biventricular (BiV) pacing (n = 711). The primary pooled analysis showed a nominally significant difference favoring MPP over BiV pacing (Mean Difference [MD]: 7.57%; 95% Confidence Interval [CI]: 0.76 to 14.39; *P* = 0.03). Because LVESV reduction was analyzed as percentage decrease from baseline, larger positive mean differences indicate greater reverse remodeling favoring MPP. However, this estimate was accompanied by substantial heterogeneity (I^2^ = 77%), and the 95% prediction interval (−6.78 to 21.92) crossed zero, indicating that the effect may not be consistent across future clinical populations. The prediction interval crossing zero indicates that future studies may show no benefit or even opposite effects, reinforcing the uncertainty of the pooled estimate despite nominal statistical significance (Fig. 3).

**Fig. 4:**
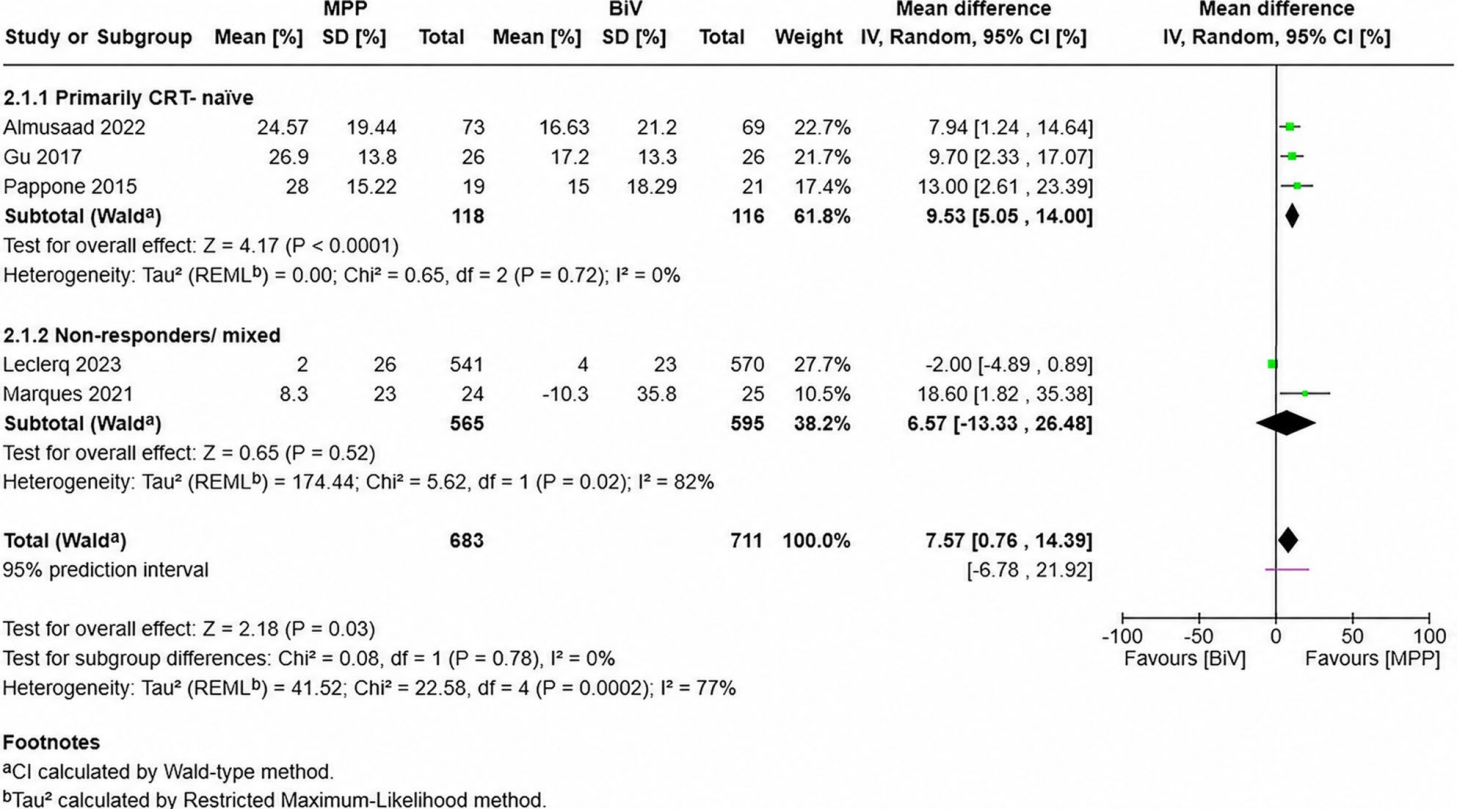
Subgroup analysis of LVESV reduction stratified by patient population: Primarily CRT-naïve patients versus prior non-responders/mixed populations. Studies are stratified into two subgroups based on the baseline characteristics of the enrolled population: (2.1.1) Primarily CRT-naïve patients (Almusaad, Gu, Pappone) and (2.1.2) prior non-responders or mixed populations (Leclercq, Marques)

To investigate the source of this heterogeneity, a sensitivity analysis was conducted by excluding the study by Leclercq et al. [7], which was the largest trial and exclusively enrolled prior CRT non-responders. This sensitivity analysis revealed a statistically significantly greater reduction in LVESV% with MPP compared to BiV pacing and resolved the heterogeneity (I^2^ = 0%). Further exploration through subgroup analysis based on patient population was performed (Fig. 4): In the subgroup of primarily CRT-naïve patients (including Pappone et al. [1], Almusaad et al. [9], and Gu et al. [5]), MPP showed a significant benefit compared to BiV pacing (MD: 9.53%; 95% CI: 5.05–14.00; *P* < 0.0001; I^2^ = 0%). In the subgroup including prior CRT non-responders or mixed populations (Marques et al. 3) [7], MPP showed a non-significant and heterogeneous effect. However, the test for subgroup differences was not statistically significant (*P* = 0.78), indicating that these findings should be interpreted cautiously. Overall, the pooled LVESV effect favors MPP on average, but the confidence in a universal benefit is limited by heterogeneity and by the prediction interval crossing zero; the signal appears more consistent in CRT-naïve patients than in prior non-responder populations. Forest plot of the LVESV analysis is shown in Figs. 3 and 4.

#### LVEF improvement

**Fig. 5:**
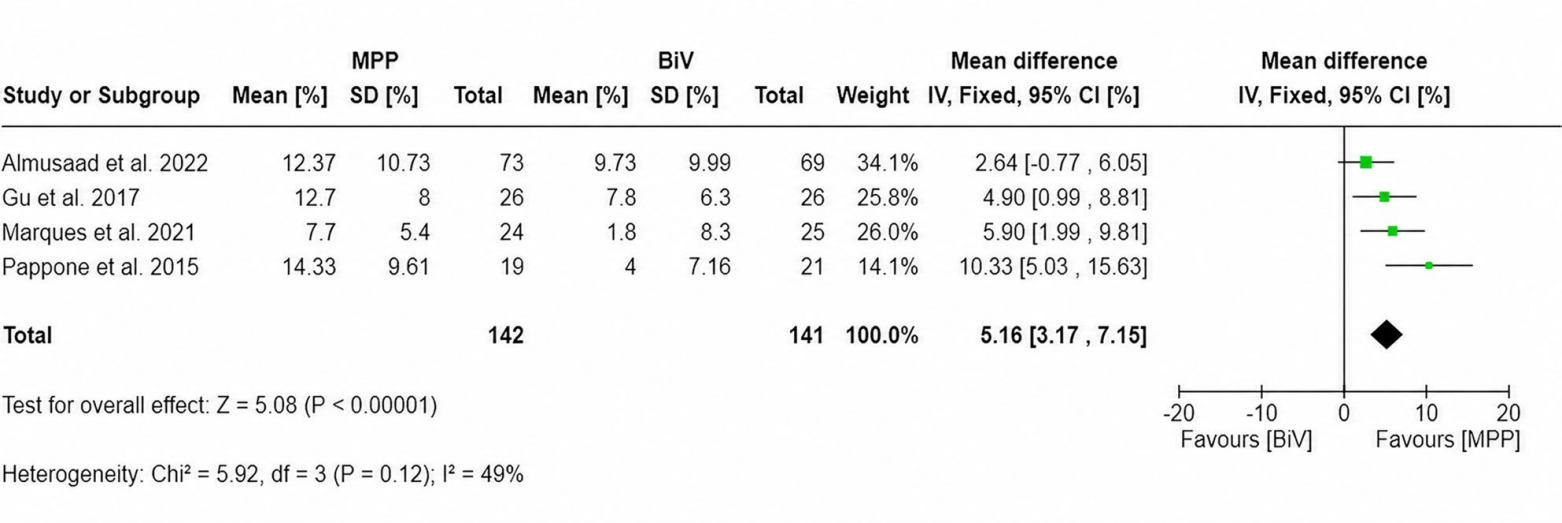
Forest plot of LVEF improvement with MPP versus BiV pacing. Comparison of LVEF improvement between MultiPoint Pacing (MPP) and Biventricular Pacing (BiV) across four studies (n = 283)

**Fig. 6:**
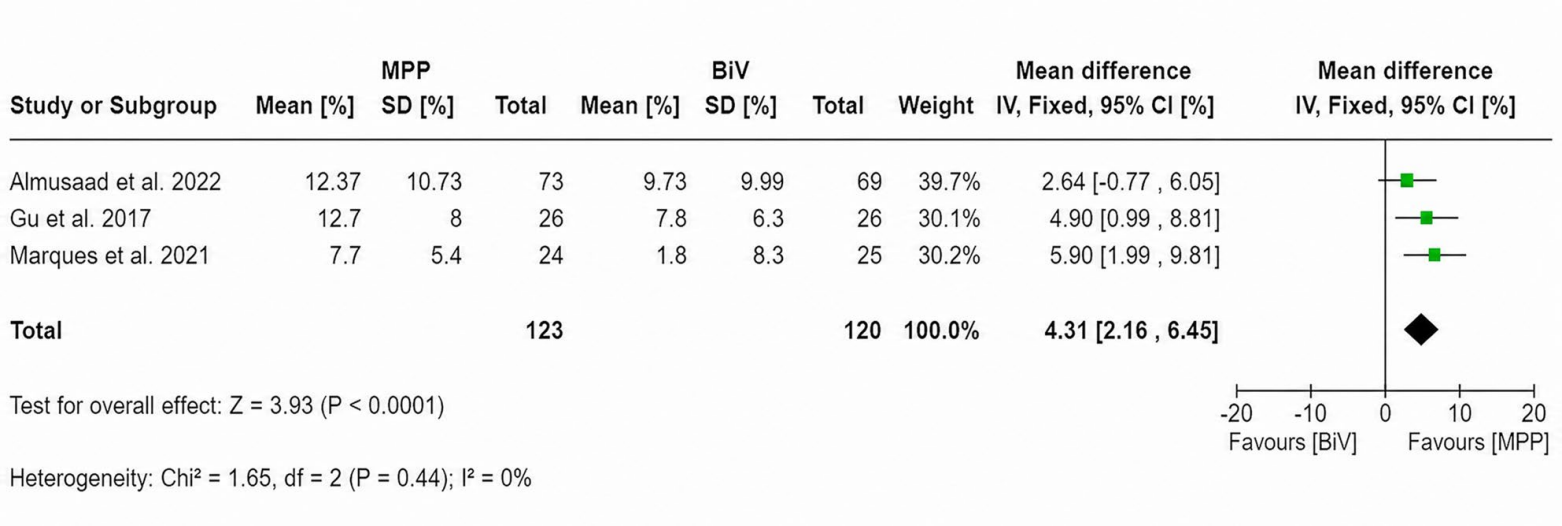
Sensitivity Analysis of LVEF Improvement (excluding outlier). Forest plot re-analyzing LVEF improvement after excluding the outlier study [1]

Four studies (n = 283) comparing MPP (n = 142) to BiV pacing (n = 141) for improvement in EF were included. The pooled analysis showed a statistically significant greater increase in EF with MPP versus BiV pacing (mean difference: 5.16%; 95% CI: 3.17–7.15; *P* < 0.00001), with moderate heterogeneity (I^2^ = 49%). Sensitivity analysis excluding the study by Pappone et al. reduced heterogeneity to zero (I^2^ = 0%) and yielded a mean difference of 4.31% (95% CI: 2.16 to 6.45; *P* < 0.0001), confirming the robustness of the finding in favor of MPP, as shown in Figs. 5 and 6.

#### Composite response rate

**Fig. 7:**
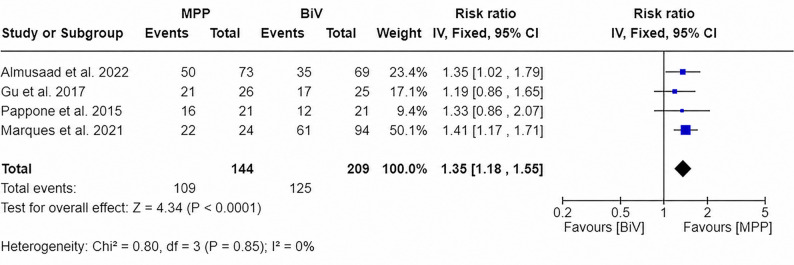
Forest plot comparing clinical response rate between MPP and BiV pacing groups. Comparison of clinical response rates between MPP and BiV pacing groups (n = 353)

Four studies (n = 353) compared MPP (n = 144) to BiV pacing (n = 209) regarding clinical response rates. The pooled analysis demonstrated a significantly higher response rate with MPP (risk ratio: 1.35; 95% CI: 1.18–1.55; *P* < 0.0001), with no heterogeneity observed (I^2^ = 0%), as shown in Fig. 7.

#### Super response rate

**Fig. 8:**
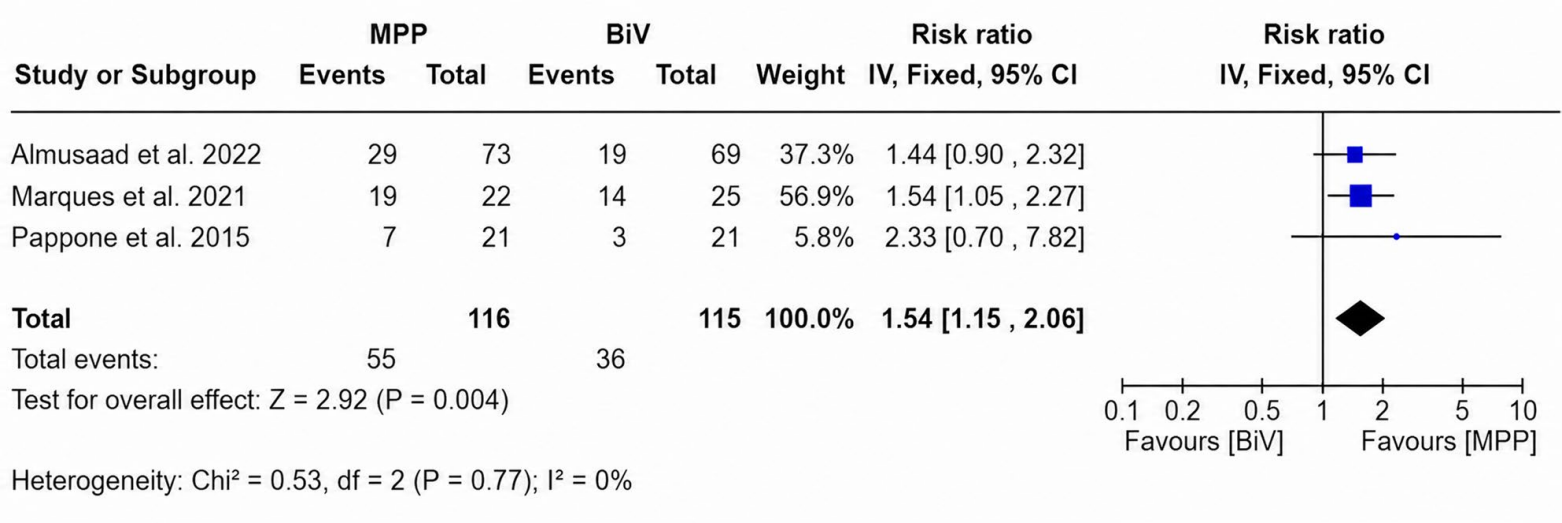
Forest plot of the super responder rate with MPP versus BiV pacing. Pooled analysis of super-response rates (defined by substantial LV reverse remodeling or ejection fraction improvement) in MPP versus BiV pacing (n = 231)

Three studies (n = 231) compared MPP (n = 116) to BiV pacing (n = 115) regarding super-responder rates. The pooled analysis showed a significantly higher super-responder rate with MPP (risk ratio: 1.54; 95% CI: 1.15–2.06; *P* = 0.004), with no heterogeneity observed (I^2^ = 0%), as shown in Fig. 8.

#### Non-responder to responder conversion rate

**Fig. 9:**
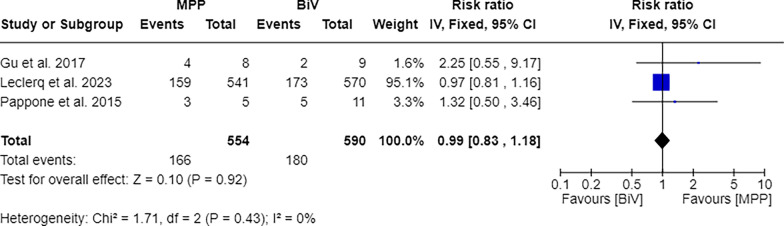
Forest plot of non-responder to responder conversion rate with MPP versus BiV pacing

Three studies (n = 1,144) compared MPP (n = 554) with BiV pacing (n = 590) for non-responder-to-responder conversion rates. In the largest trial (Leclercq et al.), the overall conversion rate was 29.4% in the MPP arm and 30.4% in the BiV arm. The pooled analysis showed no significant difference between MPP and BiV pacing (risk ratio: 0.99; 95% CI: 0.83–1.18; *P* = 0.92), with no heterogeneity observed (I^2^ = 0%), as shown in Fig. 9.

### Certainty of evidence

The certainty of the evidence for the five synthesized outcomes ranged from very low to moderate, according to the GRADE framework. LVEF improvement demonstrated moderate certainty, as all included trials consistently favored MPP with no serious imprecision, but the domain was downgraded due to concerns in the risk-of-bias assessment. The outcomes of clinical response and super-response were rated as moderate and low certainty, respectively, due to methodological limitations and small sample sizes. However, the direction of effect remained consistent. In contrast, the evidence supporting LVESV reduction and non-responder conversion was judged to be of very low certainty, primarily due to substantial heterogeneity, concerns regarding risk of bias, and wide confidence intervals that overlapped potential benefit and no effect. Overall, while the evidence indicates that MPP provides meaningful improvements in several key clinical and echocardiographic outcomes, the supporting data remain limited for outcomes that are highly variable or restricted to specific subpopulations. Importantly, the very low certainty of the evidence for LVESV reduction reinforces that the observed pooled effect should be interpreted with caution and not considered definitive evidence of benefit.

A summary of the GRADE assessment is shown in Table 4.

## Discussion

This systematic review and meta-analysis of six randomized controlled trials compared MultiPoint Pacing (MPP) with conventional biventricular pacing (BiV) in patients with heart failure. The included trials represent three distinct clinical scenarios: (i) de novo CRT implantation in CRT-naïve patients, (ii) optimization of patients who are already echocardiographic responders to conventional BiV pacing, and (iii) rescue therapy for established non-responders. The observed treatment effects appeared most consistent in the first two settings, whereas the rescue scenario was characterized by marked heterogeneity and a lack of convincing benefit. The primary pooled analysis of LVESV reduction showed a nominally significant average benefit for MPP; however, this finding should be interpreted with caution, given substantial heterogeneity and a prediction interval that crosses zero. In contrast, the secondary analyses were more favorable and more consistent, particularly for LVEF improvement and clinical response. Taken together, these findings suggest that MPP may improve reverse remodeling in selected patients, but the effect is not uniform across all CRT populations.

The observed heterogeneity in LVESV reduction was primarily driven by distinct clinical phenotypes rather than inconsistency in device efficacy. Subgroup analysis revealed that the effect of MPP appeared more consistent among CRT-naïve patients (I^2^ = 0%). In contrast, trials enrolling non-responders exhibited significant variability and showed no consistent benefit in remodeling. Sensitivity analysis confirmed this distinction; excluding the largest rescue-based trial (Leclercq et al.) eliminated the statistical heterogeneity (I^2^ = 0%), supporting the hypothesis that the remodeling effect of MPP may be more consistent when used as a de novo strategy than when applied as salvage therapy for established non-responders.

Programming strategy likely contributed to the heterogeneity observed across studies. MultiPoint Pacing can be delivered using anatomically separated vectors (MPP-AS), which activate a broader region of the left ventricle simultaneously, or with alternative programming configurations (MPP-other) [17, 18]. MPP-AS was consistently associated with better outcomes in specific populations—CRT-naïve patients and patients with wide anatomical separation (> 30 mm)—across the included trials. Also, MPP-AS considerably increased the Clinical Composite Score (CCS) [2], a validated multidimensional measure of net clinical benefit [19], in patients with larger hearts (LVEDVI > median). Furthermore, MPP-AS produced incremental improvements in LVEF, LVESV, and super-responder rates even among individuals already responding to BiV pacing, and it helped convert CRT non-responders, especially among those with high BiV pacing percentages [3]. These results demonstrate that carefully chosen programming modes and optimized anatomical lead separation are critical to MPP's efficacy.

BiV pacing percentage—defined as the proportion of beats successfully captured by biventricular stimulation—is another major determinant of MPP efficacy. Suboptimal pacing can occur in the presence of atrial tachyarrhythmias, frequent premature ventricular contractions (PVCs), or atrioventricular conduction delay [20]. In the MORE-CRT trial [7], overall conversion rates among CRT non-responders did not differ between groups; however, patients achieving > 98% BiV pacing exhibited statistically significant improvements under MPP. Similarly, in the secondary analysis by Calò et al. [21], MPP significantly reduced HF hospitalizations in patients with > 97% pacing. Collectively, these findings indicate that MPP functions as an optimization strategy rather than a salvage therapy and requires a stable, high BiV capture rate as its foundation. Consequently, clinical management should prioritize arrhythmia control and conduction optimization to secure near-complete BiV pacing before pursuing MPP programming.

Although MPP demonstrated consistent structural benefits across echocardiographic parameters, these improvements did not uniformly translate into clinical gains. Marques et al. [3] found no significant differences on the 6-min walking test, and quality-of-life outcomes were similarly variable: Varma et al. [2] observed improvement on the MLHFQ in patients with larger hearts, whereas Marques et al. [3] reported benefit only in EQ-5D scores. Functional assessment via the NYHA class was also inconsistent, reflecting both the subjective nature of the scale [22] and the paucity of the available data. Across studies, functional improvements were predominantly confined to specific subgroups, particularly CRT-naïve patients [1, 9] and pre-existing responders to BiV pacing [6]. These discrepancies underscore a structural–functional disconnect that may arise from heterogeneous assessment tools, insufficient follow-up duration, and variability in patient phenotype.

Regarding hard clinical outcomes, the MORE-CRT trial [7] showed a non-significant reduction in HF hospitalizations in the overall MPP cohort. However, when analyses were restricted to clinically enriched subgroups—such as patients with high pacing percentages in the Calò et al. [21] secondary analysis and those with larger hearts (LVEDVI > median) in Varma et al. [2]—MPP was associated with statistically significant reductions in HF hospitalizations. Mortality differences favored MPP numerically but did not reach statistical significance [7]. These neutral results are expected, as the included RCTs were designed primarily to evaluate intermediate outcomes (e.g., reverse remodeling) rather than hard endpoints, and their 6–12-month follow-up periods provide insufficient statistical power to detect differences in mortality or long-term HFH. As such, current evidence suggests that hard outcome benefits, when present, are confined to high-yield patient subgroups rather than the general CRT population.

Despite the potential clinical advantages of MPP, feasibility and battery longevity remain important practical limitations. Across studies, MPP was consistently associated with shorter projected battery life—approximately 0.9 years (11–15%) in MORE-CRT [7] and 0.44 years in Forleo et al. [23]. Earlier generator replacement increases exposure to procedural risks, including infection, lead damage, and other perioperative complications [24–26]. Feasibility challenges were also prominent, particularly for MPP-AS programming. In MORE-CRT [7], barriers such as phrenic nerve stimulation (PNS), elevated capture thresholds, and lead placement constraints resulted in MPP-AS programming failure in approximately 24% of phase 2 patients. Similar limitations were reported by Pappone et al. [1] and Gu et al. [5], in which reprogramming was often required to resolve PNS or threshold issues. Forleo et al. [27] further demonstrated that although 97% of patients were initially programmable to MPP, the feasibility fell to 87% after PNS-avoidance adjustments [28]. Importantly, aside from these feasibility and battery concerns, overall safety and adverse event rates were comparable between MPP and BiV groups. The shorter projected battery longevity associated with MPP carries implications beyond the inconvenience of earlier generator replacement. Each additional procedure exposes patients to a cumulative risk of cardiac implantable electronic device (CIED) infection, a complication associated with substantial morbidity, mortality, and healthcare costs. In CRT populations—who are often older, have multiple comorbidities, and undergo repeated device-related interventions—infection prevention strategies and individualized risk assessment are essential. Modern approaches include antibiotic-impregnated envelopes, strict aseptic protocols, and risk scores to identify high-risk candidates. Given that MPP may accelerate the replacement cycle by approximately 0.4–0.9 years, the potential incremental infection burden must be weighed against the clinical benefit, particularly in patients with an otherwise favorable baseline risk profile. Incorporating infection prevention measures and patient-specific risk assessment, as advocated in recent literature [30, 31], is therefore integral to the decision-making process when adopting MPP as a long-term pacing strategy.

Compared with the most recent prior systematic review by Mehta et al. [29], which integrated data from both randomized and observational studies, our analysis focused exclusively on RCTs and incorporated newer evidence, including the full MORE-CRT dataset [7] and the Gu et al. [5] trial. The addition of the secondary analysis by Calò et al. [21] also substantially influenced our pooled estimates. Despite methodological differences, both reviews converge in identifying considerable heterogeneity in echocardiographic response and an absence of a consistent LVESV benefit across unselected populations. However, our findings strengthen the emerging consensus that MPP should be applied as an individualized optimization therapy rather than a universal CRT enhancement strategy, with the greatest benefit expected in patients with high pacing percentage, larger ventricular volumes, or pre-existing response to BiV pacing.

### Strengths and limitations

This review has several strengths, including prospective PROSPERO registration, adherence to PRISMA guidelines [12], and rigorous appraisal of study quality using both RoB-2 [14] and GRADE [16]. The evidence base was further strengthened by the inclusion of the entire MORE-CRT dataset [7] and by sensitivity and subgroup analyses to interrogate sources of heterogeneity. However, there were limitations that posed barriers to firm conclusions, such as significant heterogeneity observed in the LVESV analysis and reliance on subgroup analyses, which are considered exploratory rather than solid findings applicable in clinical practice. Across studies, inconsistencies in response definitions, variability in reported outcomes, and limited follow-up beyond 12 months restricted our ability to evaluate long-term effects. Moreover, the definition of response differed among studies, which may introduce indirectness despite the overall consistency of effect.

## Conclusion

In summary, MPP is associated with improvements in reverse remodeling and selected clinical outcomes compared with BiV pacing. However, the LVESV findings are limited by substantial heterogeneity and a prediction interval that crosses zero; therefore, they should not be interpreted as a consistent benefit across all CRT patients. The available evidence supports MPP as an individualized optimization strategy, particularly for CRT-naïve patients and selected responder phenotypes, rather than a universal approach. Future studies should focus on identifying patient subgroups most likely to benefit.

## Supplementary Information

Below is the link to the electronic supplementary material.

Supplementary Table S1 (DOCX 4 KB)

Supplementary Table S2 (DOCX 5 KB)

## Data Availability

All data generated or analyzed during this study are included in this published article and its supplementary information files. The systematic review protocol is registered in PROSPERO (CRD420251033856).

## Abbreviations

6MWT: 6-Minute walk test
BiV: Biventricular
BMI: Body mass index
CCS: Clinical composite score
CI: Confidence interval
CRT: Cardiac resynchronization therapy
EQ-5D: EuroQol-5 dimension (quality of life scale)
GRADE: Grading of recommendations, assessment, development, and evaluations
HF: Heart failure
HFH: Heart failure hospitalization
HFrEF: Heart failure with reduced ejection fraction
LVEF: Left ventricular ejection fraction
LVEDV: Left ventricular end-diastolic volume
LVEDVI: Left ventricular end-diastolic volume index
LVESV: Left ventricular end-systolic volume
MD: Mean difference
MLHFQ: Minnesota living with heart failure questionnaire
MPP: MultiPoint pacing
MPP-AS: MultiPoint pacing using anatomically separated vectors
NYHA: New York heart association
PI: Prediction interval
PNS: Phrenic nerve stimulation
PRISMA: Preferred reporting items for systematic reviews and meta-analysis
PROSPERO: International prospective register of systematic reviews
PVCs: Premature ventricular contractions
QRSd: QRS duration
RCT: Randomized controlled trial
RoB 2: Risk of bias 2.0 tool
RR: Risk ratio
