# Supplementary material for: Efficacy of multipoint versus conventional biventricular pacing in CRT: systematic review and meta-analysis of randomized trials
Source: Egypt Heart J. 2026 Jul 9;78:53. doi: 10.1186/s43044-026-00761-4 (PMC13350621; doi:10.1186/s43044-026-00761-4)
Supplement: Supplementary file 1 — Supplementary Table S1 (DOCX 4 KB) [file 43044_2026_761_MOESM1_ESM.docx]

**Table S1**. Search strategy and results across databases

| Database | Search build | Results |
| --- | --- | --- |
| PubMed | ("Cardiac Resynchronization Therapy" OR CRT OR Resynchronization OR Pacing OR Pacemaker OR Defibrillator)  AND (Multipoint OR MPP OR Multisite OR "Multi-site" OR "quadripolar" OR "quartet")  AND (Biventricular OR BiV OR "Dual-site" OR Dualsite OR Conventional OR Standard OR traditional)  AND ("Heart Failure" OR HFrEF OR Cardiomyopathy OR "Ejection Fraction" OR Dysfunction OR "Cardiac Output" OR Hypokinesia) | 280 |
| Web of Science | ("Cardiac Resynchronization Therapy" OR CRT OR Resynchronization OR Pacing OR Pacemaker OR Defibrillator)  AND (Multipoint OR MPP OR Multisite OR "Multi-site" OR "quadripolar" OR "quartet")  AND (Biventricular OR BiV OR "Dual-site" OR Dualsite OR Conventional OR Standard OR traditional)  AND ("Heart Failure" OR HFrEF OR Cardiomyopathy OR "Ejection Fraction" OR Dysfunction OR "Cardiac Output" OR Hypokinesia) | 297 |
| Scopus | TITLE-ABS-KEY ("Cardiac Resynchronization Therapy" OR CRT OR Resynchronization OR Pacing OR Pacemaker OR Defibrillator)  AND (Multipoint OR MPP OR Multisite OR "Multi-site" OR "quadripolar" OR "quartet")  AND (Biventricular OR BiV OR "Dual-site" OR Dualsite OR Conventional OR Standard OR traditional)  AND ("Heart Failure" OR HFrEF OR Cardiomyopathy OR "Ejection Fraction" OR Dysfunction OR "Cardiac Output" OR Hypokinesia) | 270 |
| Cochrane | ("Cardiac Resynchronization Therapy" OR CRT OR Resynchronization OR Pacing OR Pacemaker OR Defibrillator)  AND (Multipoint OR MPP OR Multisite OR "Multi-site" OR "quadripolar" OR "quartet")  AND (Biventricular OR BiV OR "Dual-site" OR Dualsite OR Conventional OR Standard OR traditional)  AND ("Heart Failure" OR HFrEF OR Cardiomyopathy OR "Ejection Fraction" OR Dysfunction OR "Cardiac Output" OR Hypokinesia)  in Title Abstract Keyword | 85 |

- **Date of searching: 12/5/2025**
- **Total: 932**
- **Duplicates: 190**
- **Total after removing duplicates: 742**
