# Supplementary material for: Efficacy of multipoint versus conventional biventricular pacing in CRT: systematic review and meta-analysis of randomized trials
Source: Egypt Heart J. 2026 Jul 9;78:53. doi: 10.1186/s43044-026-00761-4 (PMC13350621; doi:10.1186/s43044-026-00761-4)
Supplement: Supplementary file 2 — Supplementary Table S2 (DOCX 5 KB) [file 43044_2026_761_MOESM2_ESM.docx]

**Table S2**: Justification of Risk of Bias Assessment of the Included Randomized Controlled Trials Using the ROB-2 Tool

| Study | Domain 1: Randomization | Domain 2: Deviations From Intended Interventions | Domain 3: Missing Outcome Data | Domain 4: Outcome Measurement | Domain 5: Reporting Bias | Overall Judgment |
| --- | --- | --- | --- | --- | --- | --- |
| Gu 2017 | **Low:** Randomization is described with balanced baseline characteristics and no evidence of allocation bias. | **Low:** Interventions delivered as assigned; device-based approach limits deviations; analyses appropriate. | **Low:** Minimal attrition, balanced across groups, unlikely to affect outcomes. | **Low:** Objective echo/device outcomes; standard methods reduce detection bias. | **Low:** All key outcomes expected for CRT studies reported; no signs of selective omission. | **Low** |
| Almusaad 2022 | **Some concerns:** Randomization used, but details on sequence generation and concealment not reported; baseline balance supports adequate implementation. | **Low:** Intervention delivery consistent; core-lab echo minimizes performance bias; ITT used. | **Low:** High follow-up completeness; missingness is rare and not outcome related. | **Low:** Blinded core-lab echo ensures objective and unbiased measurement. | **Some concerns:** Lack of protocol registration makes pre-specification unclear. | **Some concerns** |
| Leclercq 2023 | **Low:** Large multicenter RCT with registration; baseline well-balanced. | **Low:** Interventions applied per protocol; no meaningful deviations; ITT used. | **Some concerns:** A portion of patients lacked evaluable 12-month echo; although not differential, the volume of missing data introduces uncertainty. | **Low:** Core-lab, blinded assessment minimizes measurement bias. | **Low:** Registered study with prespecified endpoints; no sign of selective reporting. | **Some concerns** |
| Varma 2020 | **Low:** Based on a parent randomized trial with adequate randomization and balanced groups. | **Some concerns:** As-treated and subgroup analyses introduce some deviation from ITT and potential bias. | **High:** Substantial missing outcome data; incomplete reasons reported; missingness may relate to outcomes. | **Low:** Objective, validated outcomes assessed under blinded conditions. | **High:** Post-hoc exploratory focus without a pre-specified analysis plan increases selective reporting risk. | **High** |
| Pappone 2015 | **Low:** Randomization produced balanced baseline groups; no concerns regarding sequence or concealment. | **Low:** Protocol-driven device programming; patient and assessor blinding reduces deviations. | **Low:** Follow-up nearly complete; losses small and unrelated to treatment. | **Low:** Blinded echo assessment with consistent methodology. | **Low:** All relevant outcomes reported; no evidence of selective reporting. | **Low** |
| Marques 2021 | **Low:** Randomization clearly stated; groups balanced; no issues detected. | **Low:** Interventions delivered as assigned; participants blinded; ITT followed. | **Low:** Minimal and balanced missingness; deaths unrelated to intervention. | **Low:** Objective echo and functional assessments conducted by blinded staff. | **Some concerns:** Lack of study registration introduces uncertainty about prespecified outcomes. | **Low** |

***Table S2.*** *This table summarizes the detailed risk of bias assessment for all six randomized controlled trials included in this review, evaluated using the Cochrane ROB-2 tool. Each study was assessed across five domains: (1) bias arising from the randomization process; (2) bias due to deviations from intended interventions; (3) bias due to missing outcome data; (4) bias in measurement of the outcome; and (5) bias in selection of the reported result.
Judgements for each domain are categorized as Low risk, Some concerns, or High risk, based on methodological information reported in the original articles. The overall risk of bias reflects the highest domain-level judgement for each study. This assessment supports transparent appraisal of internal validity and informs the certainty of evidence in subsequent GRADE evaluation.*
